# Understanding of Numerical Information during the COVID-19 Pandemic

**DOI:** 10.3390/brainsci11091230

**Published:** 2021-09-17

**Authors:** Laura Zamarian, Katharina M. -A. Fürstenberg, Nadia Gamboz, Margarete Delazer

**Affiliations:** 1 Department of Neurology, Medical University of Innsbruck, 6020 Innsbruck, Austria; margarete.delazer@i-med.ac.at; 2 Faculty of Psychology, Leopold-Franzenz University of Innsbruck, 6020 Innsbruck, Austria; katharina.freiin-von-fuerstenberg@student.uibk.ac.at; 3 Laboratory of Experimental Psychology, Suor Orsola Benincasa University of Naples, 80135 Naples, Italy; nadia.gamboz@unisob.na.it

**Keywords:** numerical concepts, risk understanding, decision-making, analytical thinking, health numeracy

## Abstract

Media news during the Coronavirus Disease 2019 (COVID-19) pandemic often entail complex numerical concepts such as exponential increase or reproduction number. This study investigated whether people have difficulties in understanding such information and whether these difficulties are related to numerical competence, reflective thinking, and risk proneness. One hundred sixty-three participants provided answers to a numeracy scale focusing on complex numerical concepts relevant to COVID-19 (COV Numeracy Scale). They also provided responses to well-established objective and subjective scales, questions about affective states, and questions about the COVID-19 pandemic. Higher scores on the COV Numeracy Scale correlated with higher scores on the Health Numeracy Scale, in the Cognitive Reflection Test (CRT), and in self-assessments of verbal comprehension, mathematical intelligence, and subjective numeracy. Interestingly, scores on the COV Numeracy Scale also positively correlated with the number of consulted information sources about COVID-19. Accuracy in the CRT emerged as a significant predictor, explaining ca. 14% of variance on the COV Numeracy Scale. The results suggest that people with lower reflective thinking skills and lower subjective and objective numerical competence can be more at disadvantage when confronted with COVID-related numerical information in everyday life. These findings advise caution in the communication of relevant public health information that entails complex numerical concepts.

## 1. Introduction

The Coronavirus Disease 2019 (COVID-19) pandemic is a very exceptional event that has starkly and invasively affected several aspects of our life. It has required radical changes in our everyday habits, far-reaching behavioral adjustments, and a series of health-related decisions (e.g., wearing a mask, going into quarantine, vaccinating) [1]. In times of a pandemic, even a simple action, such as meeting a friend, may have direct detrimental consequences not only for the single individual but also for the society in general. However, how can one make a good decision without understanding the provided risk information? Many individuals cite poor information from public health authorities as a stressor during quarantine and complain about confusion [2]. Confusion may result from the lack of clear guidelines but may also be related to poor risk understanding by concerned individuals [1]. Low understanding of numerical risk information may lead, on the one hand, to unconcerned and reckless behavior and, on the other hand, to high worry and anxiety, which may also have several negative consequences [3]. It could also lead to reliance on wrong, misleading information provided in social media. This study investigated whether healthy individuals understand complex numerical concepts such as exponential increase and reproduction number, which are relevant to the understanding of COVID-19 transmission dynamics. To this aim, we developed a short ad-hoc numeracy scale.

Risk information is often conveyed through complex numerical concepts or mathematically related expressions (e.g., 20%, one out of 100, double that much, exponential increase…). It has been shown that this information is difficult to understand [4], sometimes even for highly-qualified health care professionals [5,6,7,8]. The term “numeracy” refers to the ability to understand and use numbers, perform arithmetic operations, and compare numerical magnitudes. It also concerns the ability to understand more complex numerical concepts such as probabilities, percentages, or relative risk reduction. Although it is undoubtable that humans do not act rationally as Homo economicus and that heuristics, intuitions, feelings, and beliefs play an important role in decision-making [9,10,11,12,13], there is ample evidence showing that accurate evaluation and understanding of numerical information improve risk comprehension and help make skilled, informed decisions in risk situations [14,15,16]. Higher numerical competence is associated with higher risk comprehension [17,18]. It leads to more trust in numerical information, better compliance with health instructions, a better outcome in case of chronic diseases, and even lower mortality [19]. Recently, it has been shown that higher susceptibility to misinformation about COVID-19 is associated with lower numeracy skills [20].

Previous studies showed that numeracy is poor among the general population [21]. It decreases with advancing age [22] and is low in people with low education [22,23,24], older women [22], and people with poor cognitive abilities (specifically, executive functions and mental calculation) [22,23]. In numeracy tasks, people with neurodegenerative conditions are even more at disadvantage than healthy individuals are [25,26]. In neurodegenerative patients, lower numeracy skills correlate with poorer scores in cognitive tests and a lower performance in a decision-making task simulating real-life situations [27]. Objective numeracy and subjective numeracy (i.e., the self-assessment of numerical competence) are highly correlated [28,29], so that people with high objective numeracy give, on average, high estimates of their numerical competence. Objective numeracy and subjective numeracy contribute independently to risk comprehension [24]. Increased subjective numerical ability improves performance in risk comprehension tasks through greater investment of effort and persistence [24]. 

In this study, the participants completed a paper survey containing questions about demographical information, questions about the COVID-19 pandemic, and scales for the assessment of different cognitive, subjective, and affective parameters. The participants were presented with a short numeracy scale that was developed ad-hoc and focused on complex numerical concepts relevant to COVID-19 (e.g., exponential increase, reproduction number, or growth rate; hereafter, COV Numeracy Scale). They also provided responses to well-established scales assessing objective numeracy, numerical reasoning, subjective numeracy, self-estimated intelligence, and proneness to risk. As affective states might also influence performance in numeracy tasks [24], the participants were also required to give estimates of their anxiety and depression states in the period before the pandemic as well as estimates of their current anxiety and depression states. Finally, they responded to items regarding gathering of information about COVID-19 and compliance with the prescribed containment measures. 

The primary endpoints were the relationships between the COV Numeracy Scale and other well-established objective and subjective scales. In particular, we expected that people with higher numerical competence, as defined by performance in objective numeracy and numerical reasoning tasks, would demonstrate better understanding of complex numerical concepts such as exponential increase, reproduction number, or growth rate. In line with previous studies [24,28,30], we also expected an association between better performance in numerical tasks and higher self-reported numerical competence. Whether performance in numerical tasks, specifically on the COV Numeracy Scale, is related to proneness to risk is open for investigation. The secondary endpoints were the relationships between the COV Numeracy Scale, demographic variables, affective states, the number of information sources consulted, the participants’ attitudes towards the gathered information, and their self-reported compliance with the prescribed containment measures. Previous studies showed an association between math anxiety, numerical competence, and subjective numeracy [24]. In this study, people had to estimate their general anxiety and depression levels. It may be that the experience of high anxiety levels, also when unspecific and not related to the math domain, is associated with low numerical competence on the COV Numeracy Scale. It could also be expected that people who better perform on this scale are also those who consult a larger number of information sources, state a more positive attitude towards the gathered information, and report a higher compliance with the prescribed containment measures. Finally, it is possible that performance on the COV Numeracy Scale is also related to demographic variables (age, education, sex) as found for other objective numeracy scales [22].

## 2. Materials and Methods

### 2.1. Participants

Between August 2020 and January 2021 (i.e., during the second COVID-19 “wave” in Austria and Germany), we administered a paper survey to 163 participants recruited from acquaintances and by word of mouth. The inclusion criteria were the minimum age of 18 years and the minimum education level of eight years. The exclusion criteria were history of severe neurological, psychiatric, or major medical disorders. The participants completed the survey at home. They were instructed not to use the Internet or other expedients to answer the survey.

### 2.2. Methods

#### 2.2.1. Survey

The survey contained questions about the demographical information (age, education, sex), questions about the COVID-19 pandemic, and scales for the assessment of different objective, subjective, and affective parameters. The survey was prepared for German-speaking participants living in Austria and Germany.

##### COV Questionnaire

The COV Questionnaire can be divided into the two main thematic parts: “Information about COVID-19” and “Compliance during the COVID-19 pandemic”.

The part “Information about COVID-19” contained questions requiring the participants to indicate whether they actively searched for information about COVID-19 (yes/no), how often they searched for such information (constantly, several times a day, once a day, 1–3 times a week, less often), which sources they used to get informed (e.g., radio, TV, Internet…; the participants could select multiple options), and whether they thought they were well-informed (yes, no, I do not know). The additional seven items required the participants to indicate how useful and clear they found the COVID-related information reported by the most common media (a 10-point scale ranging from, e.g., “not useful” to “very useful” was presented together with each item). We computed the median scores for these seven items for analysis purposes. Finally, the participants were required to indicate which of the 10 statements about COVID-19 were false (e.g., “the quarantine is a precautionary isolation period of 40 days” = false; “sometimes, the first clinical symptoms of the infection are fever, dry cough, and body pain” = true; seven statements were to be correctly recognized as false, three—as true). We computed the accuracy score (maximum 10) for analysis purposes. 

The second part “Compliance during the COVID-19 pandemic” required the participants to indicate on a 10-point scale ranging from “absolutely not” to “extremely” whether they adhered to the currently prescribed containment measures (e.g., wearing a mask, physical distancing, hand hygiene…) and whether they actually behaved in a more thoughtful way than before the pandemic.

##### Objective Scales

COV Numeracy Scale. We developed a multiple-choice five-item scale (in German) with questions regarding numerical concepts such as growth rate, exponential growth, or reproduction number. The questions contained terms that regularly appeared in the local mass media during the second COVID-19 “wave” in Austria and Germany. Since an expert judged the wording of one item as misleading, we removed that item and took into account only the remaining four items in our data analysis. 

We report the original questions of the COV Numeracy Scale in the Appendix A (in German) and provide a translation to the English language. Note that this version is not validated and can be only an approximate translation. Question 1 required the computation of the growth rate from the number of infected cases taking into consideration that “the growth rate halved” across the measurement points. Question 2 assessed the understanding of the concept “exponential growth”. Question 3 assessed the understanding of the concept “reproduction number of 0”. Question 4 required the computation of the “growth rate of 30%” across the measurement points.

Reflective reasoning. The Cognitive Reflection Test (CRT) [31] contains three numerical problems requiring computation of a price, the number of objects, and the number of days given distinct conditions. It assesses the ability to resist an incorrect “intuitive” response and engage in further reflection to find a numerically correct response. 

Objective numeracy. In this study, we used the extended version of the Lipkus’ Health Numeracy Scale (HNS) [21] that has been proposed by Delazer et al. [22]. On this scale, the participants are required to convert percentages, compare proportions, add or subtract a defined percentage, or demonstrate understanding of a dosage instruction as given in a short patient information leaflet. Questions (*n* = 12) are embedded in a health-related context. The HNS [21] is one of the most common scales used to assess objective numeracy. 

We used the number of correct answers on each scale for our data analysis.

##### Subjective Scales

Subjective numeracy. The Subjective Numeracy Scale (SNS, short version) [30] requires participants to indicate on a six-point scale (ranging from, e.g., “not good at all” to “extremely good”) their perceived ability to perform various mathematical tasks and their preference for the use of numerical information (*n* = 3). We computed the sum score. 

Self-estimated intelligence. In the Inventory of Self-assessed Intelligence (ISI) [32], participants indicate on a seven-point scale (ranging from −3 to +3, where 0 indicates “average intelligence”) how they estimate different intelligence dimensions. Contrary to the version by [32], we recoded the participants’ answers in order to correspond to a scale ranging from one to seven. Furthermore, we restricted our interest to four dimensions: “verbal comprehension”, “mathematical intelligence”, “memory”, and “logical thinking”. We used each single score for analysis purposes. 

Proneness to risk. The participants also provided responses to the Risk Proneness Short Scale (R-1) [33] and to the Delayed Reward scale [34]. On the R-1 scale, participants indicate on a seven-point scale (ranging from “not at all willing” to “very willing”) their willingness to take or tolerate risks (*n* = 1). The Delayed Reward scale (*n* = 12 items) assesses temporal discounting. Participants are presented with everyday situations (e.g., “When I see something I would like to have, I generally buy it whether I can afford it or not”) and are required to choose between two alternatives (“true”, coded as 1; “not true”, coded as 0). We used the sum score for analysis purposes. A lower total score indicates higher temporal discounting, i.e., higher tolerance to postpone rewards.

##### Affective Scales

By means of a self-constructed 10-point scale ranging from “not at all” to “extremely”, the participants were required to estimate their anxiety and depression states in the period before the pandemic as well as estimate their current anxiety and depression states (four items). Each score was analyzed separately. 

### 2.3. Statistical Analyses 

Statistical analyses were performed by means of IBM SPSS 26.0. Mann–Whitney tests were performed to investigate group differences (people performing at ceiling on the COV Numeracy Scale vs. others, female vs. male) in demographical variables (age, education), in objective (HNS, CRT), subjective (SNS, ISI subscales, R1, Delayed Reward Scale), and affective measures (anxiety and depression states before the pandemic, current anxiety and depression states), as well as in selected measures of the COV Questionnaire (number of information sources, accuracy in identifying true/false statements about COVID-19, compliance with the prescribed containment measures, perceived differences in behavior, estimated usefulness and clarity of COVID-related information). We opted here for nonparametric statistical methods as the subgroups starkly differed in size. We reported the Cohen’s *d* effect size in case of significant group differences. Frequency distributions (e.g., male vs. female) were compared by means of χ^2^ tests. Anxiety and depression estimates referring to the current situation were compared to the estimates referring to the period before the pandemic by means of pairwise *t*-tests. We also conducted a Pearson correlation analysis to investigate possible associations of performance on the COV Numeracy Scale with demographical, objective, subjective, and affective measures. Selected scores from the COV Questionnaire (see above) were also added into this analysis. Similarly, we investigated possible correlations for the HNS and the CRT with other measures. The measures showing a significant correlation were then entered as predictors of interest into a hierarchical regression analysis with accuracy in the COV Numeracy Scale as a dependent variable. Objective measures were entered in model 1, subjective measures—in model 2, other measures—in model 3. Significance was set at α = 0.05.

## 3. Results

The frequencies, medians, interquartile ranges, and minimum/maximum scores are reported in Table 1.

### 3.1. Demographical Information

The participants had a median age of 27.0 years (interquartile range, IQR, 24.0–47.0; minimum/maximum, 18–78) and a median education of 16.0 years (IQR, 13.0–18.0; minimum/maximum, 9–21). Fifty-nine participants were male (36.2%), 104 were female (63.8%).

### 3.2. COV Questionnaire

#### 3.2.1. Information about COVID-19

One hundred fifty-four participants (94.5%) indicated having actively searched for information about COVID-19. The frequency of accessing different information sources was as follows: five participants declared doing it constantly (3.1%), 33 participants—many times a day (20.2%), 59 participants—once a day (36.2%), 40 participants—1–3 times a week (24.5%), 17 participants—less frequently than once a week (10.4%). The people reported to use 0–6 different information sources (median, 3.0; IQR, 2.0–4.0). The majority of the participants (*n* = 125, 76.7%) stated being well-informed about COVID-19. The remaining participants stated being either not well-informed (*n* = 8, 4.9%) or not sure (*n* = 30, 18.4%). Overall, the people estimated the usefulness and clarity of COVID-related information in different media as high (median, 8.0; IQR, 6.0–9.0; minimum/maximum, 2–10). When required to identify true/false statements about COVID-19, the participants reached a median accuracy score of nine (IQR, 8.0–10.0; minimum/maximum, 0–10).

#### 3.2.2. Compliance during the COVID-19 Pandemic 

The participants reported high adherence to the currently prescribed containment measures (median, 8.0; IQR, 7.0–9.0; minimum/maximum, 0–10) and behaving more carefully than before the pandemic (median, 8.0; IQR, 7.0–9.0; minimum/maximum, 1–10). 

### 3.3. Objective Scales

**Figure 1:**
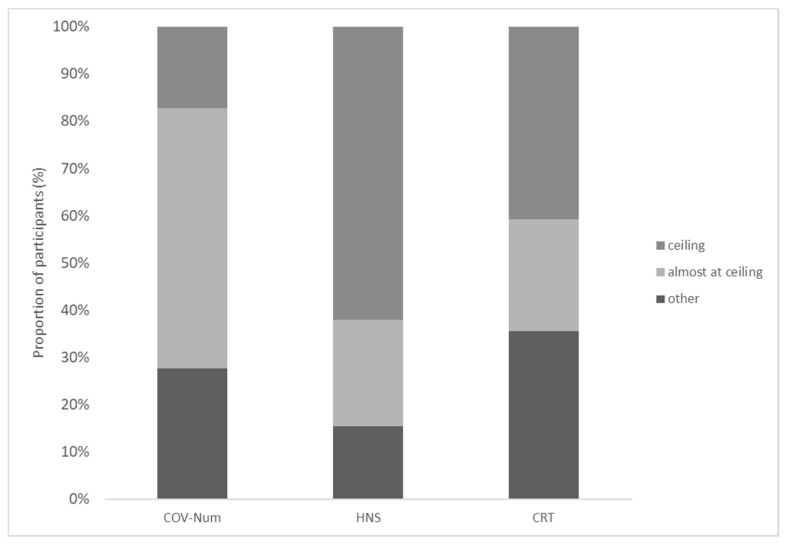
Proportion of the participants performing at ceiling, almost at ceiling, or other on objective scales. Legend: COV-Num = COV Numeracy Scale (ceiling: 4/4 correct; almost at ceiling: 3/4 correct; other: less than 3 correct); HNS = Health Numeracy Scale (ceiling: 12/12 correct; almost at ceiling: 11/12 correct; other: less than 11 correct); CRT = Cognitive Reflection Test (ceiling: 3/3 correct; almost at ceiling: 2/3 correct; other: less than 2 correct).

**Figure 2:**
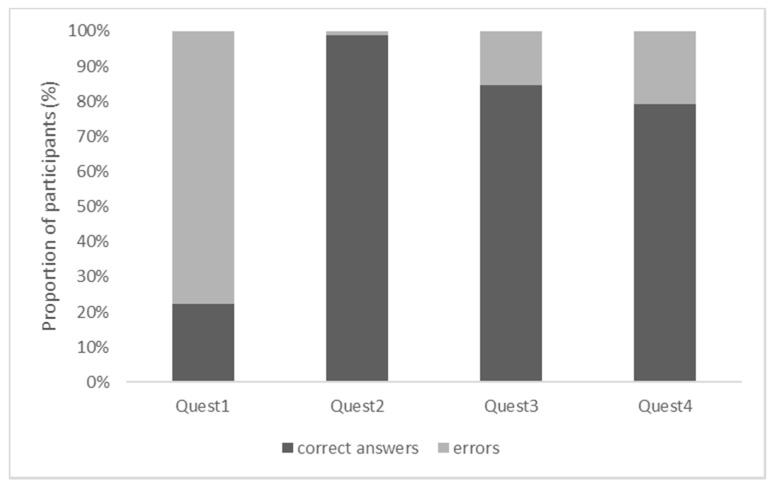
Proportion of the participants responding correctly or non-correctly to each question of the COV Numeracy Scale. Legend: Quest1 = computation of the growth rate from the number of infected cases taking into consideration that “the growth rate halved” across the measurement points; Quest2 = understanding of the concept “exponential growth”; Quest3 = understanding of the concept “reproduction number of 0”; Quest4 = computation of the “growth rate of 30%” across the measurement points.

The participants reached a median accuracy score of three on the COV Numeracy Scale, 12.0 on the HNS, and two in the CRT (Table 1). The following number of participants scored at ceiling on the COV Numeracy Scale, the HNS, and the CRT: 28 (17.2%), 101 (62.0%), and 60 (36.8%; Figure 1), respectively. Regarding the COV Numeracy Scale, the participants found questions 3 and 4 comparably difficult; question 1 was the most difficult, question 2—the easiest (χ^2^ tests, all *p* < 0.001; Figure 2).

### 3.4. Subjective Scales

The median scores on the SNS and in the ISI indicated high self-estimates of numerical competence and intelligence. Furthermore, the participants indicated low risk-taking and high resistance to immediate gratification (Table 1). 

### 3.5. Affective Scales

The median scores indicated relatively low estimates of anxiety and depression (Table 1). The estimates referring to the current anxiety and depression states were significantly higher than those referring to the period before the pandemic (*t*-tests, both *p* < 0.01).

### 3.6. Differences between People Performing at Ceiling on the COV Numeracy Scale and Others 

The people performing at ceiling on the COV Numeracy Scale scored on the HNS (z = −2.872, *p* < 0.01, *d* = 0.65) and in the CRT (z = −4.111, *p* < 0.001, *d* = 0.93) more accurately than the people not performing at ceiling. They also provided higher estimates of mathematical intelligence (z = −3.249, *p* = 0.001, *d* = 0.79) and subjective numeracy (z = −3.560, *p* < 0.001, *d* = 0.81). No other differences emerged (neither in demographical measures nor on objective, subjective, or affective scales). There were also no differences with regard to information about COVID-19 or compliance during the COVID-19 pandemic.

### 3.7. Sex Differences 

There were significant sex differences on the R1 scale (z = −2.678, *p* < 0.01, *d* = 0.45) as well as in the estimates of mathematical intelligence (z = −2.295, *p* < 0.05, *d* = 0.30), subjective numeracy (z = −2.683, *p* < 0.01, *d* = 0.37), current anxiety state (z = −2.288, *p* < 0.05, *d* = −0.32), and compliance with the currently prescribed containment measures (z = −2.325, *p* < 0.05, *d* = −0.32). Compared to the male participants, the female participants indicated lower risk-taking as well as lower estimates of mathematical intelligence and subjective numeracy. In contrast, they indicated a higher current anxiety state and higher compliance with containment measures. There were no sex differences in terms of age and education as well as on other objective, subjective, affective, or COVID-related scales.

### 3.8. Correlation Analysis 

Higher accuracy on the COV Numeracy Scale correlated with better performance on the HNS (*r* = 0.172, *p* = 0.028) and in the CRT (*r* = 0.317, *p* < 0.001) as well as with higher estimates of verbal comprehension (ISI; *r* = 0.155, *p* = 0.049), mathematical intelligence (ISI; *r* = 0.221, *p* = 0.005), and subjective numeracy (SNS; *r* = 0.260, *p* < 0.001). There was also a significant correlation between higher accuracy on the COV Numeracy Scale, a larger number of consulted information sources about COVID-19 (*r* = 0.181, *p* = 0.021), and a more positive attitude towards COVID-related information in the mass media (*r* = 0.166, *p* = 0.034). 

Higher accuracy on the HNS correlated with better performance in the CRT (*r* = 0.379, *p* < 0.001) as well as with higher estimates of subjective numeracy (SNS; *r* = 0.374, *p* < 0.001), verbal comprehension (ISI; *r* = 0.286, *p* < 0.001), mathematical intelligence (ISI; *r* = 0.329, *p* < 0.001), memory (ISI; *r* = 0.172, *p* = 0.028), and logical thinking (ISI; *r* = 0.223, *p* = 0.004). There was also a significant correlation between higher accuracy on the HNS and a higher education level (*r* = 0.174, *p* = 0.026). 

Higher scores in the CRT correlated significantly with higher subjective numeracy (SNS; *r* = 0.252, *p* = 0.001) and a larger number of consulted information sources about COVID-19 (*r* = 0.180, *p* = 0.022). Other correlations for the objective scales were not significant.

### 3.9. Hierarchical Regression Analysis 

A hierarchical regression analysis was performed, where accuracy on the COV Numeracy Scale was entered as a dependent variable. Model 1 with the HNS and CRT as predictors explained 9.2% of variance and was significant (*F*_(2,158)_ = 9.15, *p* < 0.001). Model 2, in which the SNS and estimates of mathematical intelligence and verbal comprehension were added to the Model 1 predictors, explained significantly more variance (R^2^ change = 0.050, *F*_(3,155)_ = 3.05, *p* < 0.05). The model explained 12.6% of the variance and was significant (*F*_(5,155)_ = 5.63, *p* < 0.001). Model 3, in which the number of consulted information sources and the estimates of usefulness and clarity of COVID-related information were added to the Model 2 predictors, did not explain significantly more variance. The model explained 14.1% of variance and was significant (*F*_(7,153)_ = 4.74, *p* < 0.001). In Model 3, accuracy in the CRT was the only significant predictor of performance on the COV Numeracy Scale (*p* < 0.001). The other variables were not significant.

Please note that the main results of the correlation and regression analyses did not change substantially when only the participants younger than 30 years old were included.

## 4. Discussion

This study adopted a newly developed short numeracy scale (COV Numeracy Scale) to assess the understanding of complex numerical concepts such as exponential increase, growth rate, or reproduction number. We found that less than 18% of the participants performed at ceiling on this scale. That means the majority of the participants could not understand all the complex numerical information presented. It should be noted that the COV Numeracy Scale contains numerical concepts and expressions that are regularly presented by different mass media since the beginning of the COVID-19 pandemic (at least in Austria and Germany where the recruitment of the participants took place). It seems, therefore, plausible that several individuals might have—at least to some extent—difficulties in risk understanding when confronted with this numerical information in everyday life. Interestingly, we found that people performing at ceiling on the COV Numeracy Scale differed from others in various cognitive and subjective measures. They indeed reached higher scores in the extended version of the numeracy scale proposed by Lipkus et al. [21,22] and in a test assessing reflective, numerical reasoning [31]. The people performing at ceiling on the COV Numeracy Scale also provided higher estimates of mathematical intelligence and subjective numeracy. Other differences did not emerge.

In general, better performance on the COV Numeracy Scale correlated with better performance in numerical tasks (i.e., HNS, CRT) and higher estimates of verbal comprehension, mathematical intelligence, and subjective numeracy. Interestingly, higher accuracy on the COV Numeracy Scale also correlated with a larger number of consulted information sources about COVID-19 and higher estimates of usefulness and clarity of COVID-related information in different media. In other words, the people who were more informed about COVID-19 and had a more positive attitude towards that information responded better on the COV Numeracy Scale. There were no correlations with demographical variables, affective states, measures of risk proneness and temporal discounting, accuracy in detecting true/false statements about COVID-19 (which did not entail numerical information), or compliance with the prescribed containment measures. Accuracy in the CRT emerged as a significant predictor, explaining ca. 14% of the variance in performance on the COV Numeracy Scale.

While the HNS is often taken as an objective numeracy measure [24], the CRT is mostly regarded to be a tool for assessing reflective thinking given numerical problems [16,31,35]. In this test, people are required to suppress an “intuitive” but erroneous answer and engage in further reflection to find a numerically correct response [31]. Performance in this test is thought to measure a person’s tendency towards either rapid, intuition-based processing (system I) or a more thoughtful, analytical approach (system II) [31]. People performing better in the CRT typically report a higher preference for a cognitive–reflective style [35]. Mathematical ability and rational thinking, as well as, and importantly, executive functions such as inhibition, working memory, and regulation of attention, have been found to contribute to performance in the CRT [36,37]. It has been shown that previous experience with similar numerical problems (e.g., as found on the Internet for entertainment purposes) may positively influence performance in this test [38]. It is possible that to answer the COV Numeracy Scale, individuals need cognitive abilities that are similar to those required with problems in the CRT (e.g., mathematical competence, rational thinking, and inhibition). Thus, people who rely more on reflective, analytical processes may perform better on the COV Numeracy Scale, demonstrating better comprehension of complex numerical concepts such as exponential increase or reproduction number. It should be noted that, similarly to performance on the COV Numeracy Scale, performance in the CRT correlated positively with subjective numeracy and the number of consulted information sources about COVID-19. People consulting a larger number of information sources demonstrated higher COV numeracy, higher reflective thinking, and higher subjective numeracy and stated behaving more thoughtfully than before the pandemic (these results are not reported).

Performance on the COV Numeracy Scale was also related to self-estimates of mathematical intelligence. Similarly to subjective numeracy, self-estimates of mathematical intelligence may be seen as a self-assessment of numerical competence and a measure of self-efficacy. Objective numeracy and subjective numeracy are found to be highly correlated [28,29] and contribute independently to risk understanding [24]. People with higher objective numeracy levels typically demonstrate better risk understanding [17,18] and more trust in numerical information [19]. They have more positive experiences in performing numerical tasks and higher self-efficacy. Increased subjective numerical ability is associated with greater investment of effort and persistence on challenging tasks, which also leads to improvements in performance in risk comprehension tasks [24]. Possibly, in our study, the people with higher self-estimates of mathematical intelligence were also those engaging more in solving the questions of the COV Numeracy Scale and reaching higher scores.

A growing amount of evidence has shown that factors such as education, analytical thinking, numeracy, or reflective thinking (as tested using the CRT) may play an important role in the processing of (mis-)information and in engaging in self-preserving and prosocial behaviors [20,39,40]. Regarding the COVID-19 pandemic, a recent study [20] has investigated susceptibility to coronavirus-related misinformation and its influence on the key health-related behaviors (vaccination and compliance with public health guidance such as wearing a mask in public) in large national surveys in Ireland, the USA, Spain, Mexico, and the UK. Performance in numeracy tasks and trust in scientists emerged as the most consistent predictors of decreased susceptibility to misinformation about COVID-19 [20]. Susceptibility to misinformation was associated with vaccine hesitance and reduced likelihood of complying with public health guidance [20]. Similarly, in a study carried out in March 2020 [40], people performing lower in the CRT, and thus demonstrating lower reflective thinking, were more likely to believe that the COVID-19 pandemic is a hoax and less likely to engage in helpful behaviors like social distancing and hand washing. In this study, we did not find an association between the performance on any cognitive scale (COV Numeracy Scale, HNS, and CRT) and the self-reported compliance with the prescribed containment measures. It should be noted that the people who volunteered knew that this investigation regarded the COVID-19 pandemic. Possibly, the people who were skeptical about the mainstream pandemic-related opinions or rules issued by the government were less likely to participate in the study. Therefore, the results might be “biased” with regard to self-reported compliance. Furthermore, the participants were prevalently young and had relatively high education and high numerical understanding (e.g., the median score in the expanded version of the HNS was 12 out of 12 items). The lack of a correlation might be due to low variance in the compliance scores as well as lower variance in the scores related to numerical information. Moreover, it cannot be excluded that some people adhere to pandemic restrictions because of fear and not because of high numerical understanding. The results regarding sex differences point in this direction. The female and male participants did not differ from each other in the objective numerical tasks. However, the female participants indicated a higher current anxiety state and higher compliance with containment measures than the male participants. This association should be further investigated in future studies.

We should mention some limitations. In this study, 57.1% of the survey respondents were young adults aged between 18 and 29 years old, while very few (5.6%) were older than 60 years. Furthermore, the number of male and female participants was not equal (36.2% vs. 63.8%), as well as the number of people with low (≤12 years) and high (>12 years) number of years of education (17.8% vs. 82.2%). It is therefore possible that we missed correlations between performance on the COV Numeracy Scale and demographical variables as previously found for other numeracy scales [22]. In this study, a correlation between education and objective performance was only found for the HNS, in line with previous studies [22,23]. Authors of future studies are encouraged to administer the COV Numeracy Scale to a large stratified sample of participants in order to detect possible variations in performance due to age, education level, and sex. As the items of the COV Numeracy Scale also require understanding of such terms as “reproduction number”, it could be possible that part of the variance in performance on this scale is explained by interindividual differences in semantic knowledge. Tasks tapping on semantic/encyclopedic knowledge in addition to tasks assessing number processing and mathematical computation might help highlight possible difficulties with COVID-related numerical information.

## 5. Conclusions

Media news often require the understanding of complex numerical concepts such as exponential increase, reproduction number, or mortality rate. The results of this study suggest that some individuals may have—at least to some extent—difficulties in risk understanding when confronted with this numerical information in everyday life. People with lower numeracy, lower self-efficacy, and/or lower reflective thinking skills are those who are more at disadvantage in this regard. Although these results are preliminary, they suggest caution in the communication of relevant public health information that entails complex numerical concepts.

## Figures and Tables

**Table 1:** Frequencies, medians, interquartile ranges, and minimum/maximum scores in the administered scales.

	Mdn	P.25	P.75	Min.	Max.
COV Numeracy Scale (total correct)	3.0	2.0	3.0	1	4
HNS (total correct)	12.0	11.0	12.0	6	12
CRT (total correct)	2.0	1.0	3.0	0	3
SNS (sum)	15.0	13.0	16.0	6	18
ISI					
Verbal comprehension	5.0	5.0	6.0	3	7
Mathematical intelligence	5.0	4.0	6.0	1	7
Memory	5.0	4.0	6.0	2	7
Logical thinking	5.0	5.0	6.0	2	7
Risk Proneness Short Scale (R-1)	4.0	3.0	5.0	0	7
Delayed Reward Scale (sum)	3.0	2.0	5.0	0	11
States prior to the pandemic					
Anxiety	2.0	1.0	3.0	1	8
Depression	2.0	1.0	3.0	1	10
Current state					
Anxiety	3.0	2.0	5.0	1.0	9.0
Depression	2.0	1.0	3.0	1.0	9.0

Legend: HNS = Health Numeracy Scale; CRT = Cognitive Reflection Test; SNS = Subjective Numeracy Scale (short version); ISI = Inventory of Self-estimated Intelligence; Mdn = median; P.25 = 25th percentile; P.75 = 75th percentile; Min. = minimum; Max. = maximum.

## Data Availability

The data are available from the authors upon reasonable request.

## Appendix A. COV Numeracy Scale (German)

1. In der Stadt X konnte die Wachstumsrate infizierter Menschen halbiert werden. Hier sehen Sie drei Messergebnisse für drei verschiedene Standorte. Über welchen Ort sprechen wir?
  (a) 100—200—100
  (b) 100—200—300
  (c) 100—200—150
2. Was bedeutet exponentielles Wachstum? Welche Zahlenreihe wächst exponentiell?
  (a) 2—4—8
  (b) 100—200—300
  (c) 2000—4000—6000
3. Ein Replikationsfaktor von 0 bedeutet:
  (a) Es werden immer gleich viele Leute infiziert.
  (b) Es werden 10x so viele Leute infiziert.
  (c) Es wird niemand mehr infiziert.
4. Die Zahl der Erkrankungen erhöht sich täglich um 30%. Welche Zahlenreihe entspricht diesem Wachstum?
  (a) 1000—1300—1600
  (b) 100—130—169
  (c) 10—30—90

### COV Numeracy Scale (Approximate Translation in English)

1. In the town X, the growth rate of infected people halved. Here, you see three different points of measurement for three different places. About which location are we speaking?
  (a) 100—200—100
  (b) 100—200—300
  (c) 100—200—150
2. What does exponential growth mean? Which row of numbers does exponentially grow?
  (a) 2—4—8
  (b) 100—200—300
  (c) 2000—4000—6000
3. A reproduction number of 0 means:
  (a) The same number of people is always infected.
  (b) Ten times as many people are infected.
  (c) Nobody is infected anymore.
4. The number of infections increases daily by about 30%. Which row of numbers does correspond to this growth?
  (a) 1000—1300—1600
  (b) 100—130—169
  (c) 10—30—90

The correct answers are as follows: b, a, c, b.
